# Development of an Ultrasensitive Impedimetric Immunosensor Platform for Detection of *Plasmodium* Lactate Dehydrogenase

**DOI:** 10.3390/s19112446

**Published:** 2019-05-29

**Authors:** Yu Kong Low, Jianxiong Chan, Gita V. Soraya, Christelle Buffet, Chathurika D. Abeyrathne, Duc H. Huynh, Efstratios Skafidas, Patrick Kwan, Stephen J. Rogerson

**Affiliations:** 1 Department of Medicine, The Peter Doherty Institute for Infection and Immunity, The University of Melbourne, 792 Elizabeth Street, Melbourne, VIC 3000, Australia; low.u.kong@gmail.com (Y.K.L.); Kristel.buffet@gmail.com (C.B.); 2 Department of Medicine, Royal Melbourne Hospital, The University of Melbourne, Victoria 3050, Australia; jianxiong.chan@unimelb.edu.au (J.C.); gitavitasoraya@gmail.com (G.V.S.); 3 Department of Biochemistry, Faculty of Medicine, Hasanuddin University, Makassar 90245, Indonesia; 4 Department of Electrical and Electronic Engineering, Melbourne School of Engineering, The University of Melbourne, Victoria 3010, Australia; chathurika.abeyrathne@unimelb.edu.au (C.D.A.); duchau.huynh@gmail.com (D.H.H.); sskaf@unimelb.edu.au (E.S.)

**Keywords:** immunosensor, biosensor, impedance, ultrasensitive, malaria, pLDH

## Abstract

Elimination of malaria is a global health priority. Detecting an asymptomatic carrier of *Plasmodium* parasites to receive treatment is an important step in achieving this goal. Current available tools for detection of malaria parasites are either expensive, lacking in sensitivity for asymptomatic carriers, or low in throughput. We investigated the sensitivity of an impedimetric biosensor targeting the malaria biomarker *Plasmodium* lactate dehydrogenase (pLDH). Following optimization of the detection protocol, sensor performance was tested using phosphate-buffered saline (PBS), and then saliva samples spiked with pLDH at various concentrations. The presence of pLDH was determined by analyzing the sensor electrical properties before and after sample application. Through comparing percentage changes in impedance magnitude, the sensors distinguished pLDH-spiked PBS from non-spiked PBS at concentrations as low as 250 pg/mL (*p* = 0.0008). Percentage changes in impedance magnitude from saliva spiked with 2.5 ng/mL pLDH trended higher than those from non-spiked saliva. These results suggest that these biosensors have the potential to detect concentrations of pLDH up to two logs lower than currently available best-practice diagnostic tools. Successful optimization of this sensor platform would enable more efficient diagnosis of asymptomatic carriers, who can be targeted for treatment, contributing to the elimination of malaria.

## 1. Introduction

Malaria, an acute febrile illness caused by *Plasmodium* parasites that are spread through the bites of infected female Anopheles mosquitoes, caused 435,000 deaths in 2017 alone [1]. Of the five parasite species that infect humans, *Plasmodium falciparum* and *Plasmodium vivax* are the most common; *P. falciparum* causes the majority of malaria-related mortalities, while *P. vivax* is the most widely distributed malaria parasite globally [2].

Since 2000, global efforts have led to a substantial decline in malaria episodes and deaths, and an increasing number of countries have moved from malaria control to malaria elimination, which the World Health Organization (WHO) defines as the interruption of local human malaria transmission for three consecutive years [3]. Recently, efforts to eliminate malaria appear to be stalling [1]. To meet the unique challenges posed by malaria elimination, the Malaria Eradication Consultative Group on Diagnoses and Diagnostics (malERA) and the WHO Evidence Review Group on Malaria Diagnosis in Low Transmission Settings highlight the need for improved diagnostic tools with high analytical sensitivity, the ability to differentiate species, high throughput, and low cost [4,5].

In countries approaching elimination, there is generally a high proportion of asymptomatic and often very low-density infections. A strategic shift from passive case detection to active screening will be required to achieve prolonged interruption of transmission [6,7]. Asymptomatic, submicroscopic infections can harbor gametocytes that may infect mosquitoes [8,9]. Submicroscopic infections are defined as being below the lower limit of detection (LLOD) of light microscopy (LM), the gold standard for clinical diagnosis of malaria, which is around 50–100 parasites/μL under field conditions [10,11,12]. This limit is also below the sensitivity of established Rapid Diagnostic Tests (RDTs), which use immunochromatographic assays to detect parasite proteins in blood [13,14]. Although submicroscopic infections can be less transmissible by mosquitoes [9], at low transmission levels approaching malaria elimination, submicroscopic infections predominate and they can be the source of 20–50% of human-to-mosquito transmissions [9]. This important reservoir of infection needs to be targeted for elimination.

LM and RDTs are the current gold standards for clinical diagnosis of malaria. WHO guidelines dictate that patients with suspected clinical malaria episodes should undergo at least one of the two tests prior to administration of antimalarial treatment [15]. While sufficiently sensitive for identification of symptomatically infected people (moderate- to high-density infection), LM underestimated the population prevalence of *P. falciparum* by roughly fifty percent on average [16]. Similarly, RDTs also significantly underestimate the prevalence of infection [17,18]. This is more profound in populations with lower parasite densities [16]. The low sensitivity of these two current point-of-care tests highlights the need for more sensitive point-of-care diagnostic tools. Currently, the most commonly targeted malaria antigens for RDTs are Histidine-Rich Protein 2 (HRP-2) and *Plasmodium* lactate dehydrogenase (pLDH). HRP-2 expression is only found in *P. falciparum* [19], while pLDH is common across all human-infecting *Plasmodium* species [20]. Other options for malaria detection are Nucleic Acid Amplification-based Techniques (NATs), such as PCR, loop mediated isothermal amplification (LAMP), and quantitative nucleic acid sequence-based amplification. However, while highly sensitive [21,22], NATs are currently infeasible for mass deployment due to a combination of a slow turnover rate, high upfront and per-sample costs, and difficulty of deployment in resource-limited environments. Attempts to adapt NATs for field application have yet to lead to operational deployment [23,24].

A detection method as convenient as LM or RDT, that has the sensitivity of NAT, will help to push toward malaria elimination. Impedimetric biosensors are promising options to help close current diagnostic gaps, due to their high sensitivity, low cost, and amenability to miniaturization. They detect interactions in attached bioreceptor components through measuring changes in electron transfer resistance. Biosensors can be conjugated with selective antibody, which increases its selectivity and sensitivity, especially for small molecules [25]. These sensors have demonstrated high levels of sensitivity and specificity for label-free detection of various targets, including nucleic acids and proteins [26,27,28]. A review of impedimetric biosensors found the LLODs to frequently reach low picogram/mL ranges [29]. In terms of antigen concentration, LLODs of RDTs for malaria antigens would fall within the range of 4 ng/mL for HRP-2 and 45 ng/mL for pLDH [30], multiple logs higher than the theoretical limits of impedimetric biosensors.

This study reports the performance of impedimetric biosensors targeting pLDH as a proof-of-concept of the potential for impedimetric biosensors to overcome the existing limitations of LM and RDTs. Here, the sensor platforms consisted of anti-pLDH antibodies chemically cross-linked to interdigitated electrodes (IDEs)-based impedimetric biosensors. First, we optimized the detection protocol and conducted proof-of-concept experiments using pLDH in phosphate-buffered saline (PBS) and then assessed the performance of the protocol on human saliva samples.

## 2. Material and Methods

### 2.1. General Design

**Figure 1:**
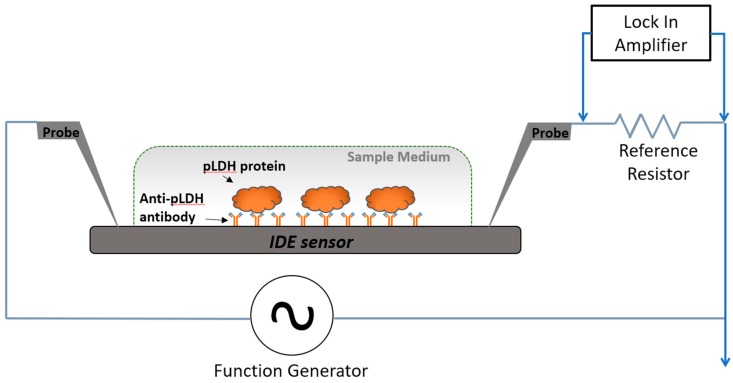
Circuit model and measurement setup used for detection of *Plasmodium* lactate dehydrogenase (pLDH). The cross-sectional view of the measurement setup illustrates detection of spiked pLDH protein in a sample medium with the sensing area. Probe electrodes were placed to deliver excitation current to, and to measure electrical signals from, the sensors.

The general design of the platform comprised an IDE functionalized with an antibody targeting pLDH. Binding of pLDH to the antibody resulted in changes in the signal impedance of the IDE sensor. These changes were detected by a lock-in amplifier (Figure 1). The IDE sensors were fabricated via UV-lithography, and have previously demonstrated high sensitivity for label-free detection of various biomolecules [26,27,28]. The study was approved by The University of Melbourne ethics committee (ethics ID 1443204.4).

### 2.2. pLDH and Anti-pLDH Antibody

Recombinant *P. vivax* LDH antigen was purchased from CTK Biotech (San Diego, CA, USA). Monoclonal pan-malaria anti-pLDH antibody was purchased from Vista Diagnostics (Kirkland, WA, USA).

### 2.3. Fabrication and Functionalization of IDE Sensor

The IDE sensors were fabricated at the Melbourne Centre for Nanofabrication (Clayton, Victoria, Australia) using UV-lithography on BOROFLOAT glass wafers (University Wafer, Massachusetts, USA). The sensor design and fabrication method has been described previously [26,27,28]. Wafers were cleaned with isopropyl alcohol and coated with hexamethyldisilazane (HMSD) (MicroChemicals GmbH, Ulm, Germany) at 3000 rpm for one minute and AZ1512HS (MicroChemicals GmbH, Ulm, Germany) photoresist at 3000 rpm for one minute before undergoing baking at 100 °C for 90 s (SUSS Delta 80RC). AZ1512HS is the photoresist, while HMSD aids the adhesion of AZ1512HS to the silicon wafer. A chrome mask of the sensor design was applied on the substrate and was followed by ultraviolet (UV) exposure (75 mJ/cm^2^). A thin film of chrome (5 nm), gold (100 nm), and titanium (5 nm) was then deposited on the substrate, followed by a lift-off process to reveal the IDE pattern. Each IDE sensor consisted of paired electrode arrays with a finger length of 980 µm, a finger width of 8 µm, and a gap width of 8 µm. The sensor was subject to additional SiO_2_ deposition (25 nm thickness) through e-beam evaporation (Intlvac Nanochrome™ II, Fort Collins, CO, USA). The fabricated sensors were washed with acetone, isopropyl alcohol, and H_2_O, and dried under nitrogen gas. Sensors were then plasma-treated (PE-25 Plasma Etch, Carson City, NV, USA) with argon (75%) and oxygen (25%) for 5 min at 50 W power and a 30 cc/min flow rate. The treated sensors were functionalized in accordance with our established protocols [26,27,28]. The sensors were incubated in filtered (Corning^®^ 0.2 μm) 2% 3-Aminopropyl) triethoxysilane (APTES) (Sigma Aldrich, Saint Louis, MO, USA) in an ethanol solution for 1 h, followed by 3 × 5-min washes in 100% ethanol with gentle shaking. This allowed for salinization of the activated sensor surfaces. The sensors were then submerged in filtered (0.2 µm filter) 2.5% glutaraldehyde in milli-Q water for 2 h, and then washed three times in milli-Q water for 5 min each time before being left to dry in a fume hood. This process created free aldehyde groups on the sensor surfaces. Anti-pLDH antibodies were then incubated on the sensing area at 100 µg/mL at 4 °C overnight. After incubation, slides were washed with PBS and incubated for 1 h in 1% ethanolamine (Sigma Aldrich, Missouri, USA) to block any unreacted aldehyde groups before being washed and stored in PBS until use.

### 2.4. pLDH Detection Assay

The sensors were incubated with 15 µL of PBS or saliva spiked with pLDH at relevant concentrations and incubated at room temperature for one hour. For the experiments with saliva, one part protease inhibitor (100× P8340 Protease Inhibitor Cocktail, Sigma Aldrich, Missouri, USA) per one hundred parts of saliva was added prior to addition of pLDH or saliva. The incubation time was increased to two hours. After incubation, the sensors were washed three times in PBS for 5 min each time and left to dry in a fume hood.

### 2.5. Electrical Measurement

Impedance of the sensors was measured using our previously established circuit setup [28,31], in which the sensor is represented as a resistor (R) in series with a capacitor (C) connected in series with a 1 kΩ reference resistor (*R_ref_*). A function generator provided the input sinusoidal alternating current (AC) excitatory signal (*V_in_*) at 20 mV peak-to-peak voltage (*V_pp_*) at multiple frequencies (10 kHz, 20 kHz, and 50 kHz). Properties of the sensors were first measured using a lock-in amplifier, which records the amplitude of the output voltage (*V_out_*) and phase across the *R_ref_*, first post functionalization to obtain baseline measurements, and then measured again after sample incubation. The output voltage (*V_out_*) and phase across the *R_ref_*, was then used for the acquisition of frequency (*ω*) dependent impedimetric parameters, including impedance magnitude (|Z|), capacitance (*C*), and resistance (*R*), using the following equation [27,28]:

*V_out_*/*V_in_* = *R_ref/_*(|Z| + *R_ref_*)

|Z| = R − j/(*wC*)

### 2.6. Statistical Analysis

MATLAB^®^ was used to extract the sensor impedance magnitude. The baseline impedance values were used to determine outliers in accordance with our previously developed method [28].

The magnitude and percentage of change in impedance (∆|Z|, % ∆|Z|) between sample and baseline impedance are described throughout the results section, unless otherwise stated.

All plots and statistical analysis were generated by GraphPad PRISM 7. Outliers were first identified and removed using the ROUT method (GraphPad PRISM). Next, Welch’s *t*-tests were used for all experiments that only compared two conditions, while ordinary one-way ANOVA with a Dunnett’s multiple comparison test (single pooled variance set to determine the degree of differentiation against the non-spiked negative control) were used for experiments comparing three or more conditions. *p*-values ≤ 0.05 were considered statistically significant.

## 3. Results

### 3.1. Feasibility of pLDH Detection on IDE Sensor

IDE sensors functionalized with anti-pLDH antibody were incubated with either pLDH-spiked (2.5 ng/mL) PBS or the non-spiked control at room temperature for 1 h. The sample was then subjected to electrical reading at 10 kHz, 20 kHz, and 50 kHz.

**Figure 2:**
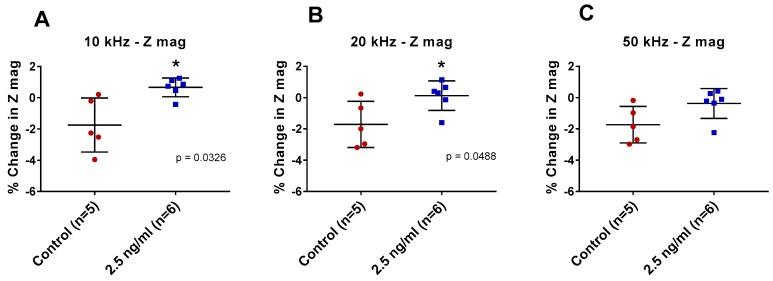
Percentage changes in impedance magnitude (Z mag) with pLDH. Functionalized interdigitated electrode (IDE) sensors were incubated with 2.5 ng/mL pLDH in phosphate-buffered saline (PBS) and non-spiked PBS at measurement frequencies (**A**) 10 kHz, (**B**) 20 kHz, and (**C**) 50 kHz. Error bars are standard deviation, * indicates *p* ≤ 0.05 (Welch’s *t*-tests).

There was a significant difference between pLDH-spiked samples and non-spiked samples at 10 kHz and 20 kHz (*p* = 0.0326 and *p* = 0.0488, respectively) but not at 50 kHz (Figure 2). Based on these findings, 10 kHz was arbitrarily selected as the measurement frequency for future experiments as it showed greater change in impedance. Using these criteria, further experiments were conducted to determine the lower detection limit of the sensor platform for pLDH in PBS.

### 3.2. Detection Range of pLDH in the IDE Sensor

**Figure 3:**
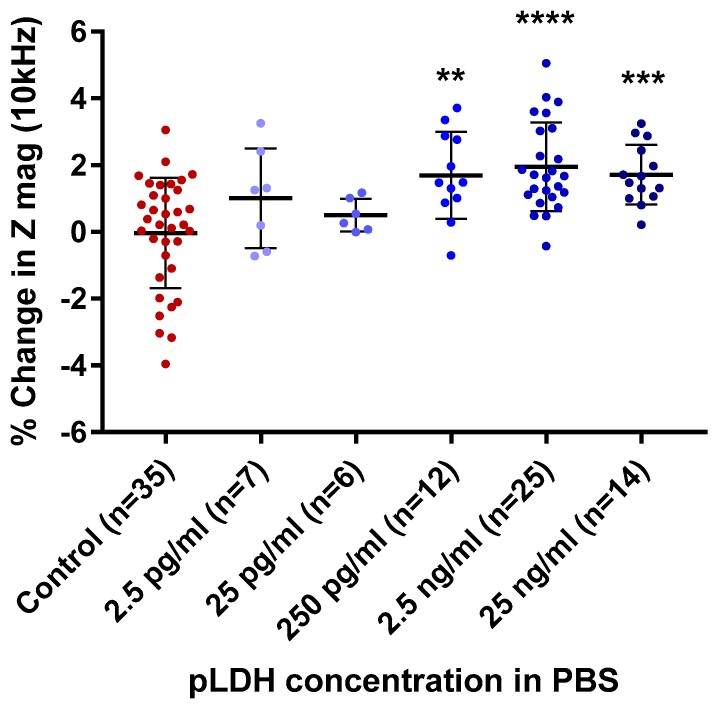
Aggregate data of percentage changes in impedance magnitude (Z mag) with increasing concentrations of pLDH in PBS. Percentage change impedance of with non-spiked PBS (control), 2.5 pg/mL, 25 pg/mL, 250 pg/mL, 2.5 ng/mL, and 25 ng/mL of pLDH, at a measurement frequency of 10 kHz. Error bars are standard deviation, ** *p* ≤ 0.01, *** *p* < 0.001, **** *p* < 0.0001 compared to control (Dunnett’s test).

After demonstrating the feasibility of pLDH detection, we next determined the detection range of pLDH, specifically the lowest detection limit, by testing a range of concentrations. Figure 3 shows the percentage changes in impedance magnitude at 10 kHz following incubation of PBS spiked with pLDH at concentrations from 25 ng/mL to 2.5 pg/mL in steps of 10-fold dilution.

Significant differences in percentage changes in impedance magnitude were seen between the non-spiked PBS blank and the pLDH-spiked PBS at pLDH concentrations of 250 pg/mL, 2.5 ng/mL, and 25 ng/mL (*p* = 0.0016, *p* < 0.0001, and *p* = 0.0007, respectively). There was no significant difference at pLDH concentrations of 25 pg/mL and 2.5 pg/mL. There was overlap between the ranges of percentage change in impedance magnitude of the non-spiked and spiked PBS at all concentrations of pLDH. The results suggest that the LLOD of the sensors for pLDH in PBS lies between 25 pg/mL and 250 pg/mL.

### 3.3. Detection of pLDH in Saliva by IDE Sensors

Following the experiments using PBS, the protocol was used to detect pLDH in saliva. Human saliva was spiked with 2.5 ng/mL of pLDH and subjected to electrical measurement at 10 kHz.

**Figure 4:**
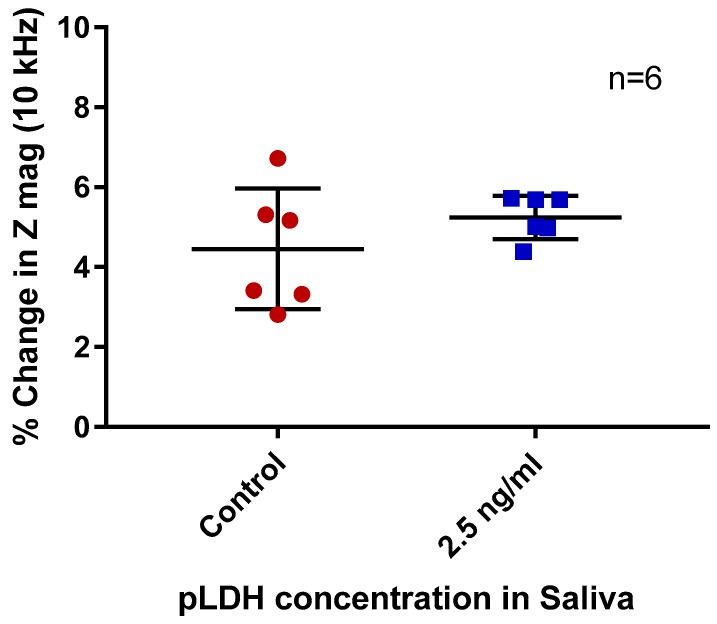
Percentage changes in impedance magnitude (Z mag) of non-spiked (control) and pLDH-spiked saliva. Measurement frequency 10 kHz. Error bars are standard deviation, *n* = 6 per group.

When saliva spiked with pLDH was compared to non-spiked paired saliva (Figure 4), no significant difference in percentage change in impedance was observed (*p* = 0.257). A generally larger percentage change in impedance compared to PBS samples was also observed.

## 4. Discussion

The two current standard tests for malaria diagnosis, LM and RDTs, have relatively low sensitivity, which limits their utility for malaria elimination. On the other hand, NAT, which has a higher sensitivity of detection is currently infeasible for mass deployment due to factors such as slow turnover rate and high upfront and per-sample test costs. Therefore, there is a need for more sensitive and simple diagnostic tools for the detection of low-density parasitemia, which will be key to malaria elimination. This study presented a novel way of measuring pLDH via impedimetric biosensors, setting a basis for a platform with potential of detecting infections with substantially lower parasite densities than the current LM and RDTs.

The feasibility of detecting pLDH on an IDE sensor was first determined. This was done by comparing the impedance characteristics between pLDH-spiked PBS and non-spiked control on IDE sensors functionalized with anti-pLDH antibody. The sensor showed different impedance characteristics between samples spiked with pLDH and blank, suggesting that the sensor can detect spiked pLDH. Among the range of excitation frequencies applied (10 kHz, 20 kHz, and 50 kHz), 10 kHz was found to elicit the most significant difference in the change in impedance magnitude between samples (Figure 2A). This frequency was used for all subsequent experiments.

As the detection was done on a relatively high concentration of pLDH (2.5 ng/mL), the next step was to determine the detection limit for this sensor. This was done with a range of pLDH concentrations to determine the range of detection of this sensor. The sensor platform showed that it could successfully detect concentrations of pLDH in PBS as low as 250 pg/mL (Figure 3). As there were no significant changes at 25 pg/mL, the LLOD for this setup was likely to be between 25 pg/mL and 250 pg/mL (Figure 3). Future work focusing on pLDH concentrations between this range will allow the LLOD to be determined. Nonetheless, the detection concentration of 250 pg/mL is approximately two logs lower than the currently available RDTs (Table A1) [30]. It also falls into the detection range for malaria elimination [4]. The detection, however, is not concentration-dependent, as the percentage change in impedance (Z mag) did not increase with increasing pLDH concentration (250 pg/mL to 25 ng/mL). A potential reason is that the amount of pLDH detected is limited to the amount of antibody functionalized on the sensor’s surface. It is possible that, at 250 pg/mL, all available antibodies on the sensor surface had been coupled with pLDH; thus, additional pLDH were not detected. The experimental conditions established in this part of the study served as a proof-of-concept for pLDH detection and formed the basis for the next experiment where pLDH was spiked into saliva.

In this part of the study, the work was translated from the conceptual experiments in the artificial solvent (PBS) to a biological solvent that is a better approximation of operational conditions. Impedance measurements showed a generally larger percentage change in impedance in both spiked saliva and blank saliva as compared to PBS samples. No significant change was observed with pLDH-spiked saliva. This lack of significance could be due to the presence of non-specific binding of salivary proteins and interference with binding or detection due to physical and chemical properties of saliva. A study investigating the total protein content of saliva suggested that the salivary protein content of the average human lies within the range of 0.72–2.45 mg/mL [32]. Even at the lower end of the range, the concentration of salivary protein is approximately five logs higher than the concentration of spiked pLDH. Given the substantial total protein content of saliva, and its absence in PBS alone, there is a high chance of non-specific proteins binding onto the sensor, interfering with specific detection and diminishing its ability to detect pLDH. This is a general issue with saliva-based sensors and a limitation in this study [33,34]. Future work should focus on ways to overcome this limitation. This may include sensor modification and sample processing. Sensor modification will aim to increase sensitivity of the sensor to the target protein; modification will include increasing blocking intensity of the sensor and increasing the concentration of antibody functionalized on the sensor surface. For sample processing, surfactant, such as Tween-20, can be added to the sample. This may increase the specificity of the target.

Another general limitation of our approach is the relatively high variation in measurements among replicates within and between experiments, which is reflected in the overlapping ranges of the percentage change in the impedance magnitude between samples. This might be due to fabrication and manual functionalization processes that take place over multiple days and involve multiple steps. These sources of variation could be reduced in the hypothetical end-product through automation of the fabrication and functionalization processes.

## 5. Conclusions

This work demonstrated the proof-of-concept that an IDE sensor platform could detect a concentration of pLDH (in PBS) as low as 250 pg/mL, which falls into the detection range suitable for malaria elimination. While further optimization is required to prepare the sensors for use in conjunction with biological substrates, several potential avenues were identified for further research to move forward.

## Appendix A

**Table A1:** Comparison of lower limit of detections (LLODs) of current best-practice malaria diagnostic tools.

Method	LLOD *	Reference
Light Microscopy	~320 ng/mL	[10,14]
RDTs	25 ng/mL	[13]
This work (in PBS)	250 pg/mL	

* lower limit of detection. Abbreviations: PBS phosphate-buffered saline; RDTs, rapid diagnostic tests.
